# Color-coded visualization of magnetic resonance imaging multiparametric maps

**DOI:** 10.1038/srep41107

**Published:** 2017-01-23

**Authors:** Jakob Nikolas Kather, Anja Weidner, Ulrike Attenberger, Yannick Bukschat, Cleo-Aron Weis, Meike Weis, Lothar R. Schad, Frank Gerrit Zöllner

**Affiliations:** 1 Department of Medical Oncology and Internal Medicine VI, National Center for Tumor Diseases, University Hospital Heidelberg, Heidelberg, Germany; 2 Computer Assisted Clinical Medicine, Medical Faculty Mannheim, Heidelberg University, Mannheim, Germany; 3 Institute of Clinical Radiology and Nuclear Medicine, University Medical Center Mannheim, Heidelberg University, Mannheim, Germany; 4 Institute of Pathology, University Medical Center Mannheim, Heidelberg University, Mannheim, Germany

## Abstract

Multiparametric magnetic resonance imaging (mpMRI) data are emergingly used in the clinic e.g. for the diagnosis of prostate cancer. In contrast to conventional MR imaging data, multiparametric data typically include functional measurements such as diffusion and perfusion imaging sequences. Conventionally, these measurements are visualized with a one-dimensional color scale, allowing only for one-dimensional information to be encoded. Yet, human perception places visual information in a three-dimensional color space. In theory, each dimension of this space can be utilized to encode visual information. We addressed this issue and developed a new method for tri-variate color-coded visualization of mpMRI data sets. We showed the usefulness of our method in a preclinical and in a clinical setting: In imaging data of a rat model of acute kidney injury, the method yielded characteristic visual patterns. In a clinical data set of N = 13 prostate cancer mpMRI data, we assessed diagnostic performance in a blinded study with N = 5 observers. Compared to conventional radiological evaluation, color-coded visualization was comparable in terms of positive and negative predictive values. Thus, we showed that human observers can successfully make use of the novel method. This method can be broadly applied to visualize different types of multivariate MRI data.

Multiparametric magnetic resonance imaging (mpMRI) is used to measure several physical parameters for each spatial location in a patient. In the clinic, this technology has important applications: for example, it is used to diagnose prostate cancer according to the PI-RADS criteria^1^. Usually, these physical parameters are visualized as gray scale images or one-dimensional color coded images and human observers visually evaluate these images successively or side-by-side^2^. Because this procedure yields many independent images, examining multi-channel datasets can be very laborious. For example, to read a typical MRI data set of the prostate according to the current PI-RADS criteria approximately takes 20 minutes^3^.

Grayscale visualization is the most common way of displaying radiological measurements in clinical routine. This way of visualizing data disregards color. However, humans have evolved in a colorful environment and color is an extremely important carrier of information in many aspects of life^4^. Not surprisingly, color is a highly efficient carrier of information and humans can quickly spot visual targets based on color^5^,^6^. Color processing in the human brain is a complex process that involves many different parts of the nervous system^7^. This machinery enables humans to distinguish millions of colors^8^. In contrast, humans can only distinguish approximately 720 different gray shades on typical medical displays^2^.

The aim of data visualization is to make objective information accessible to the human mind^9^. Grayscale intensity coding is one possible carrier for this information – but the above-mentioned studies show that color is potentially a much better carrier of information. In the context of imaging, it follows that human observers can potentially extract more information from color-coded images than from grayscale images. This increase of transmittable visual information constitutes an *information gain*. This idea is the basis for a new visualization method for mpMRI data that we present in this paper.

MpMRI data sets consist of several spatially aligned channels. By using a color-coded representation, this information can theoretically be merged into one image. A commonly used approach to combine several image channels in one image is to overlay a functional measurement on top of a structural CT or MRI image^10^. However, this approach cannot be used to visualize more than two channels at once. A simple way of visualizing more than two channels is to use the RGB color space that is used in computer displays: to encode tri-variate imaging data, each parameter map is assigned to one of the basic display colors red, green, blue. However, this method leads to distortions of the data and is prone to introduce artifacts, caused by the pronounced nonlinearity of the human perception of colors in the RGB color space^11^,^12^.

For computed tomography (CT) imaging, several studies have assessed the use of color transfer functions and their impact on observer performance^13^,^14^. For MRI data, published approaches include 3D visualization^13^, glyph-based methods^15^ and color coded visualization of temporal differences in dynamic contrast-enhanced (DCE) MRI breast imaging data^16^. Yet, to our knowledge, there is no color-coded visualization technique available that can be readily applied to three-channel, spatially aligned mpMRI data sets.

In this study, we present a possible solution to these limitations of previous visualization methods. We propose a new method for simultaneous color-based visualization of up to three spatially aligned image channels. First, we describe the development of a tri-variate color map that is adapted to the human visual system. Second, we explore how this color map can be used to process routine MRI data sets. Third, we assess diagnostic performance of blinded observers in a typical diagnostic setting in the clinic.

## Material and Methods

### Reproducibility and ethics statement

All source codes used for this study are available under the MIT license (http://opensource.org/licenses/MIT) and can be accessed via the following DOI: 10.5281/zenodo.208185. All experimental measurements can be accessed as a raw data via this DOI: 10.5281/zenodo.205081. Anonymized DICOM datasets of all samples shown in the figures can be accessed via this DOI: 10.5281/zenodo.205089. All experiments were carried out in accordance with the Declaration of Helsinki and were approved by the institutional ethics board (2013-824R-MA). The institutional ethics board waived the need for informed consent for this retrospective analysis of fully anonymized samples. Animal data were retrieved from a previously published study^17^. All animal procedures were performed according to the Guide of the Care and Use of Laboratory Animals published by the National Academy of Sciences and were approved by the local authorities (Regional council Karlsruhe, G40/10).

### Data sets

As a preclinical example, we used a data set of perfusion MRI data of experimental kidney injury in rats as described before^17^. Briefly, measurements were performed on a 3 T scanner (Tim trio, Siemens Healthcare Sector, Erlangen, Germany) using an eight channel receive-only volumetric rat array (RAPID Biomedical GmbH, Rimpar, Germany) for signal detection. DCE-MRI was performed using a 3D time-resolved angiography with stochastic trajectories (TWIST) sequence (16) with the following parameters: TR/TE/FA = 3.4 ms/1.4 ms/20°, matrix = 192 × 84, FOV = 114 × 50 mm^2^, a GRAPPA factor of 2 and 28 slices. The nominal temporal resolution was 0.9 s per volume. Images were continuously acquired for 6 minutes resulting in 400 volumes. After the 15^th^ volume, 0.05 ml of contrast agent (Dotarem, Guerbet, France) was manually administered in the femoral vein, followed by a 1.0 ml saline flush. Of the acquired data the parametric maps of the Plasma flow, Plasma Volume and Tubular flow were calculated using an in house certified OsiriX plugin (UMMperfusion 1.5.2)^18^,^19^.

As a clinically relevant example, we used mpMRI measurements of prostate cancer patients as described by Weidner *et al*.^3^. Briefly, the prostate multi-parametric data sets consist of a T2-weighted image, apparent diffusion coefficient (ADC) map, a diffusion weighted image with b = 800, and prostate blood flow (PBF) map. Imaging was performed at a 1.5 T scanner (Magnetom Avanto, Siemens Healthcare, Erlangen, Germany) with parameters are given in Table 1. ADC maps were reconstructed inline at the scanner while PBF maps of the prostate were calculated using the above mentioned perfusion plugin. Clinico-pathological characteristics of the sample collective are given in Table 2.

### Computational implementation

All computational methods in this study were implemented in MATLAB (MATLAB R2015b, Mathworks, Natick, MA, USA) if not otherwise noted. They were run on a standard computer workstation (2.2 GHz Intel Core i7, 16 GB RAM). All visual experiments were performed on a Macbook Pro Retina Display (Apple, Cupertino, CA) using the Adobe RGB 1998 color profile. Colored MRI image were saved as DICOM files and were viewed with OsiriX Viewer (Pixmeo Sarl, Bernex, Switzerland).

### Tri-variate color-based reconstruction

**Figure 1:**
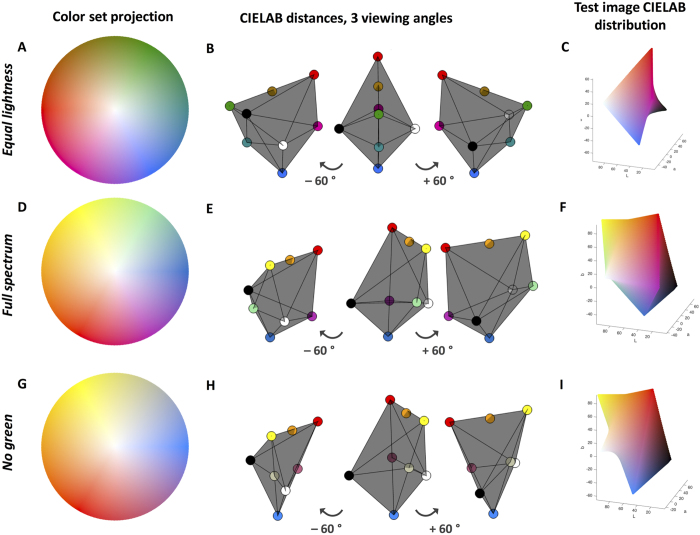
Characteristics of tri-variate color maps. (**A**) 2D projection of the “equal lightness” color map. (**B**) 3D-CIELAB view of the anchor points: It can be seen that the color map is symmetric in the perceptual color space so that no distortions are introduced in the data. (**C**) Pixel color distribution in a test image that was created by using this color map. (**D–F**) Corresponding views for an alternative color map (“Full spectrum”). (**G–I**) Corresponding views for another alternative color map (“No green”).

Color maps were constructed in a linear, perceptually uniform manner in CIELAB color space similarly to our previously published method^20^. By using the CIELAB color space, we ensured that Euclidean distances of signal intensities linearly corresponded to color differences in terms of human perception^21^. We constructed a three-dimensional color map with eight anchor points: 1^st^ “no signal in any channel” (black), 2^nd^ “maximum signal in all channels” (white), 3^rd^–5^th^ “no signal in two channels, maximum signal in one channel” and 6^th^–8^th^ “maximum signal in two channels, no signal in the third channel”. All other elements of the color map were linearly interpolated from these anchor points. The following constraints were applied: 1) Black (L = 0, A = 0, B = 0) and white (L = 100, A = 0, B = 0) should be the most extreme points on either sides (1^st^ and 2^nd^ anchor point). 2) The other anchor points (3^rd^ to 8^th^) should be part of the L = 50 plane. 3) The volume of the polyhedron should be large in order to include many different colors. These constraints guaranteed optimal characteristics of the three-dimensional color map. It can be seen that the resulting color map is symmetric in CIELAB space (Fig. 1A–C). We used the following anchor points (colors given as RGB hexadecimal codes). 1^st^ black (#000000), 2^nd^ white (#FFFFFF), 3^rd^ red (#F40000), 4^th^ green (#009100), 5^th^ blue (#1173FE), 6^th^ magenta (#EB009C), 7^th^ cyan (#008B8E), 8^th^ dark orange (#A27200). This color map is shown in Fig. 1A–C, while Fig. 1D–I show characteristics of two alternative color maps that do not satisfy all of the above-mentioned criteria. However, in special circumstances, these alternative color maps might have other benefits. For example, the color map shown in Fig. 1G–I could be better suited for observers with deuteranomaly because it does not contain green hues.

The color map in Fig. 1A–C was used to encode tri-variate data. Initially, the assignment of image channels to anchor points of this color map was arbitrary – in other words, the color map could be arbitrarily rotated. However, it is known that reddish visual targets are easier to spots than targets of different colors. This holds true even for desaturated targets, showing that reddish hues can be efficiently used to encode visual cues that are of interest^22^. Therefore, we rotated the color map in such a way that in the prostate cancer data set, cancerous tissue regions mapped to reddish areas of the three-dimensional color map.

### Data preprocessing and postprocessing

Before combining the images to a color-coded visualization, each channel was preprocessed as follows: (1) images were resampled to be spatially aligned to the T2 image (by using the native OsiriX resampling functionality), (2) all pixels with an intensity below the 1st quantile or above the 99th quantile were considered as outliers and were removed, (3) the channels were normalized to a range of 0 to 1, and (4) contrast within each channel was maximized by stretching the histogram so that 1% of the data was maximally saturated. After creating the merged colored image, the result was overlaid to the axial T2 image to allow better co-localization of anatomical structures. This procedure was applied to the prostate data set. For the rat kidney data set, all pixels below 2% or above 98% were removed. The rat data set was acquired with a clinical 3 Tesla system and dedicated coils and presented more noise and thus more outliers than the prostate data set, which was acquired with a clinical 1.5 Tesla MRI. Also, for the rat data, the following modifications were made to this procedure: no T2-overlay was created and the data were already spatially aligned in this application so that no resampling was necessary.

### Measuring perceptual distance

After applying the new visualization procedure to prostate MRI data sets, we investigated whether tumorous areas took on different colors than non-tumorous areas. Based on the histopathological annotation, we manually delineated the largest contiguous tumor area and the largest contiguous non-tumor area in a representative axial image. We calculated the mean color of the tumor area in CIELAB space. Then, for each image pixel, we calculated the Euclidean distance to the mean tumor color in CIELAB space. Because distances in the CIELAB space are proportional to perceived color differences, this distance represented the perceptual difference (or perceptual contrast) for each pixel to the mean tumor color.

### Observer study

**Figure 2:**
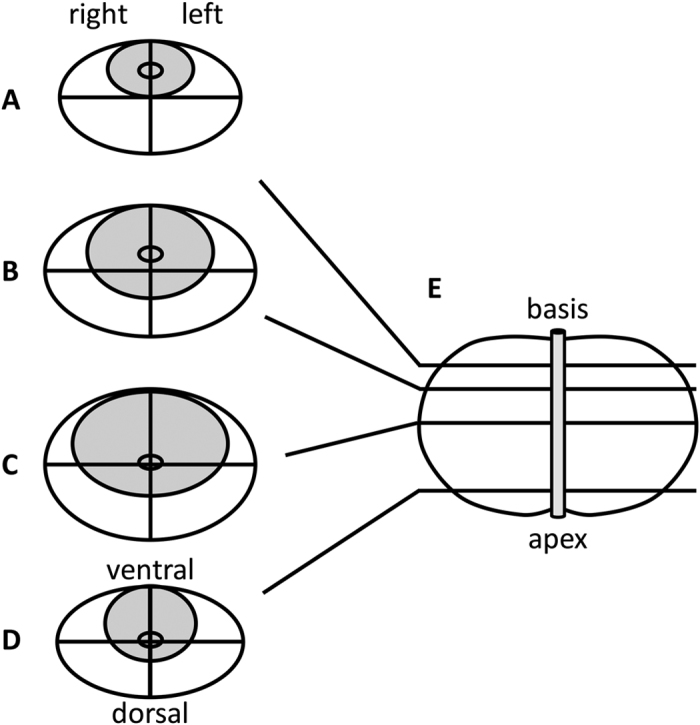
Partitioning of the prostate for the user study. Here, it is shown how the prostate was divided into segments for radiological and histopathological evaluation. (**A–D**) the four axial cuts that were considered, (**E**) sagittal view of the prostate and urethra.

In a previous study by Weidner *et al*.^3^, conventional expert reading of MR images (before total prostatectomy) was compared to systematic histopathological evaluation of these samples (after total prostatectomy). Primary end point was the detection of cancer in each segment of the prostate. In each prostate, 28 segments were defined as follows: 4 transversal planes were considered and each plane was divided in 8 sectors, except for the highest plane, which was only divided in 6 sectors (Fig. 2). In the present study, we used the same partitioning when presenting the color-coded visualizations to human observers. One expert observer (attending physician in radiology) and four radiological trainees (three residents within radiological training programs, one specialized medical intern) participated. Each observer evaluated each segment of the prostate and decided whether cancer was present or not. For each segment, this decision was compared to the histopathological gold standard and positive predictive value (PPV) and negative predictive value (NPV) were calculated. Before the actual experiment, each observer received a short training (approx. 10–15 minutes) and was explained the theory behind the visualization approach. Also, he or she was shown one example of a prostate mpMRI data set before and after the visualization procedure (this example was not included within the analyzed set).

## Results

### Application to perfusion MRI in a preclinical animal model of kidney injury

**Figure 3:**
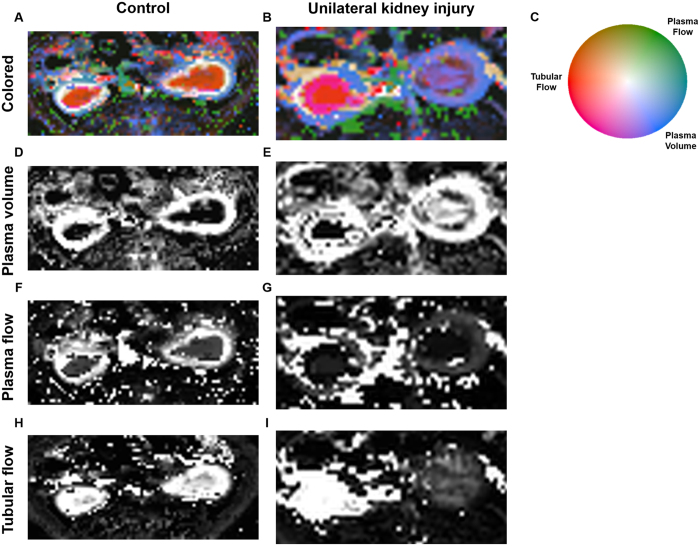
Example of perfusion MRI data sets of rat kidneys. (**A**) Control rat kidneys, tri-variate visualization of tubular flow (red), plasma flow (green), plasma volume (blue). It can be seen that both kidneys show a regular stratified pattern with high tubular flow present in the renal medulla and high plasma volume present in the renal cortex. (**B**) Perfusion MRI after temporary ligature of the left renal artery (on the right-hand side in the image). It can be seen that after kidney injury, the regular pattern is disrupted while it is preserved in the contralateral kidney. These changes are well visualized by a tri-variate color map. (**C**) Color map.

We applied the new visualization technique to a preclinical and a clinical data set. First, we chose a preclinical model of acute kidney injury in the rat and used three perfusion parameters: Tubular flow, plasma flow and plasma volume (Fig. 3). We found that in control animals, kidneys typically showed a very regular stratified pattern (Fig. 3A) that was disrupted after kidney injury (Fig. 3B). These changes were well visualized by a tri-variate color map while in conventional gray-scale images, this pattern was not as easily visualized because each parameter was shown in a separate image (Fig. 3D–I).

### Color-coded images can be used to visualize prostate cancer in MRI data

**Figure 4:**
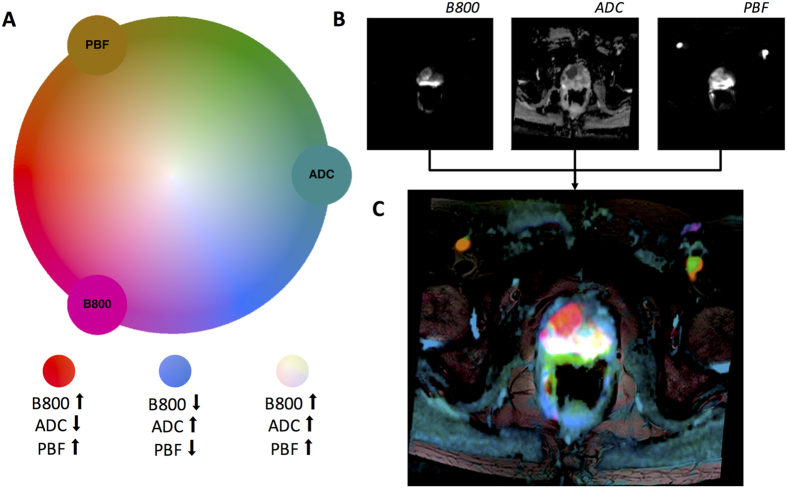
Color-based reconstruction of a multiparametric data set. (**A**) Equal luminance color map containing three variables (2D projection): PBF (Prostate blood flow), ADC (apparent diffusion coefficient) and B800 (diffusion parameter at 800 ms. Each color in the map corresponds to a unique combination of parameters. (**B**) Underlying parametric maps. (**C**) Reconstructed image. A carcinoma in the prostate can be seen as a red patch.

Second, we applied the method to a clinically relevant case and used mpMRI measurements of perfusion and diffusion in patients with suspected prostate cancer (Fig. 4C). In these data sets, tumors are generally hyperintense in the b800 image, hypointense in the ADC map and hyperintense in the PBF map. Correspondingly, in the multivariate visualization, prostate tumors took on reddish hues while surrounding prostate tissue took on blue-greenish hues.

**Figure 5:**
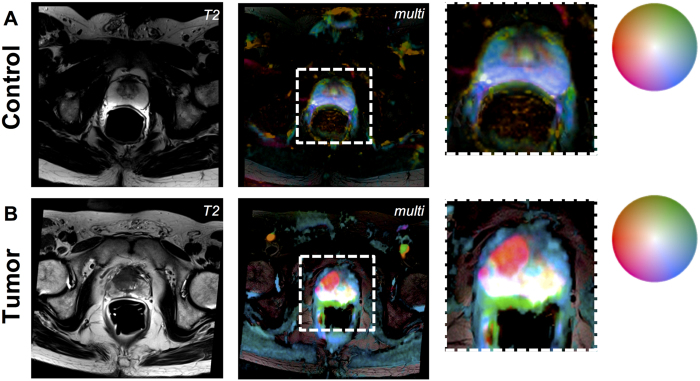
Examples of a control prostate and prostate cancer. (**A**) Patient with no evidence of prostate cancer (left: T2-weighted image, middle: multi-colored visualization of perfusion and diffusion imaging, right: enlarged detail). (**B**) Prostate cancer patient, panel layout corresponding to **A**.

**Figure 6:**
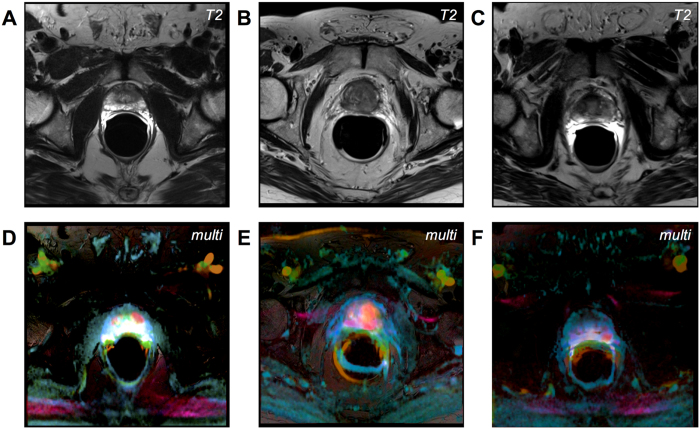
More examples of prostate cancer patients. (**A–C**) T2-weighted images. (**D–F**) corresponding multivariate visualization. Prostate tumors take on a red hue.

This color map was applied to mpMRI datasets of patients with suspected prostate cancer. In Fig. 5A, the result can be seen for a control patient with no evidence of prostate cancer. In Fig. 4B, a patient with histological evidence of prostate cancer is shown. The tumor lights up as a red patch whereas normal prostate tissue takes on blue-greenish hues or white. Comparable examples are shown in Fig. 6.

### Perceptual contrast between prostate tumors and non-tumorous prostate areas

**Figure 7:**
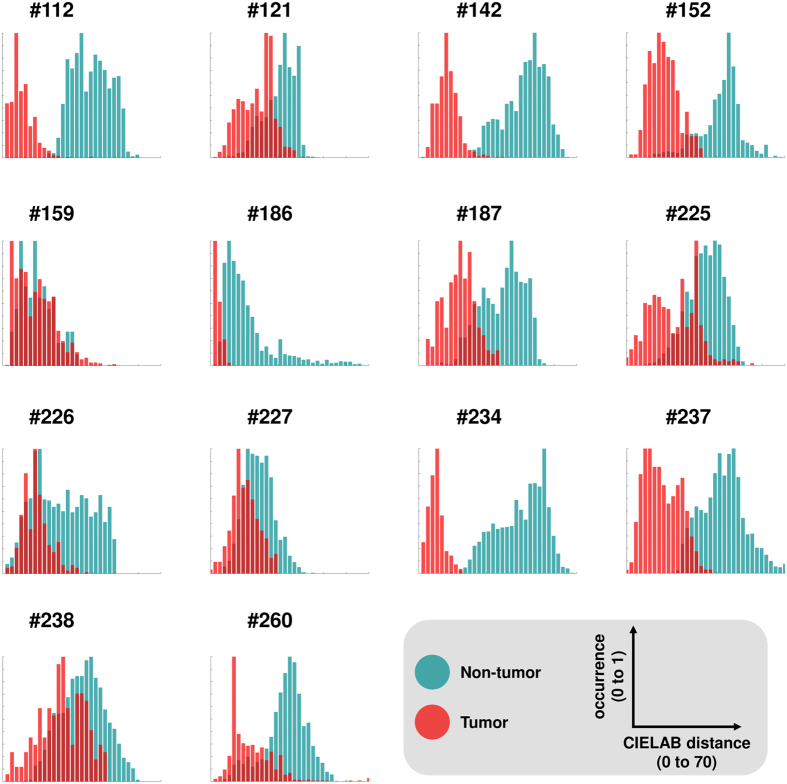
Measurements of perceptual contrast in prostate mpMRI data sets. This figure shows perceptual contrast between tumor vs. non-tumor areas in color-coded prostate mpMRI data sets. Each panel corresponds to one of N = 14 patients. For each patient, the largest contiguous tumor area and the largest contiguous non-tumor area was manually delineated. The mean color of the tumor in CIELAB space (L, A, B coordinates) was calculated and used as a reference point. Then, for each pixel in the tumor and non-tumor area, the Euclidean distance to this reference point was calculated. Because of the way the CIELAB space is calibrated, these distances represent perceptual differences for human observers. The distribution of these distances is shown as a histogram: red for all pixels in the tumor area and blue/green for all pixels in the non-tumor area. It can be seen that in all N = 14 patients, the median contrast for non-tumor to the reference point is higher than for tumor. This shows that in principle, it is possible to discern tumor and non-tumor areas solely based on color.

When comparing the histopathological annotation to the color-coded prostate data sets, it was evident that reddish tumorous areas could be discerned from the blue-greenish background. To quantify this subjective observation, we measured perceptual contrast between tumor and non-tumorous areas. As a reference point, we used the mean color of the tumor in CIELAB space. Then, in each of our N = 14 samples (including the training sample), we measured the perceptual difference from the reference point to all pixels in tumor and non-tumor areas. We found that the median perceptual distance from tumorous areas to the mean tumor color was lower than for non-tumorous areas. This was the case in all 14 samples, as can be seen in Fig. 7. These experiments show that it is theoretically possible to visually discern tumor vs. non-tumor based on the color. Next, we went on to validate this finding in a real-world setting.

### Observer performance is comparable to conventional data reading

To quantify the diagnostic accuracy that can be achieved with the proposed method, we performed an observer study with N = 5 human observers at different stages of their training. We compared their performance (measured as PPV and NPV) against an expert reading of the conventional visualization and against a systematic histopathological workup of N = 13 cases. We found that PPV and NPV were comparable in all groups: For the conventional readings, we re-evaluated raw data from a previous study^3^ and found a PPV of 0.77 and NPV of 0.65 for the presence of prostate cancer in a given prostate segment. Using the new color-coded visualization, an expert (N = 1) achieved a PPV of 0.75 and an NPV of 0.65. The average diagnostic accuracy of radiological trainees (N = 4) was reflected in a PPV of 0.76 and an NPV of 0.61. Table 3 summarizes sensitivity, specificity, PPV and NPV for all observers and Table 4 contains the raw contingency data. In summary, all these values are comparable indicating that the novel color-coded visualization enables both expert and trainee readers to detect prostate cancer with sufficient accuracy.

## Discussion

### Benefits of the proposed method

Here, we have presented a new method for the color-coded visualization of mpMRI data sets. Conventionally, only a one-dimensional color space is used to map signal intensities to pixel intensities in MRI images. For the first time, our study extends this to a three-dimensional color space. In an environment of ever-growing amounts of data, there is a real need of new data analysis techniques. Our new approach addresses this need, could now be used to explore other advanced visualization techniques and could stimulate research in this field.

Applied in a preclinical and in a clinical setting, our method can be used to visualize three independent channels from a mpMRI data set as one image. Our computational experiments and the observer study demonstrate that this type of visualization indeed enables human observers to discern clinically relevant pathologies.

Normally, a multiparametric data set is visualized as several independent grayscale images that are shown successively or side-by-side – or one channel is overlaid on the other by using an arbitrary one-dimensional color map. Using our method, the whole data set can be read by viewing one combined color-coded image that is based on a perceptually optimized color map. Conceptually, this is a major improvement because the visual system of human observers can be used more efficiently. Thus, more information can be made available for clinical or basic research-related insight based on imaging data.

We envision that our proposed method could be used in clinical routine as a plugin for radiology image viewers. As soon as radiologists gain experience with this new type of visualization, they might use it in a wide range of applications. One possible benefit of the method would be to get a quick initial impression of an imaging data set without having to look at different MRI sequences in parallel. Also, a possible benefit would be to provide intuitive visualizations for non-radiologists or patients. Also in a scientific setting, our method could be used to enable an easy-to-understand visualization of multiparametric measurements.

### Training effects

Pattern recognition by human observers requires experience^23^,^24^. Especially, the detection of prostate cancer in MR images significantly improves with training^25^. Consequently, this new way of visualization can probably only be exploited completely after a training period. In the user study that we present, observers received only an extremely short training session – in fact, they were only shown one example of a prostate that contained cancerous and non-cancerous regions. Yet, these observers were already able to detect cancer tissue with a classification performance that was comparable to the gold standard. Possibly, this performance would further improve after a longer training period. The novel visualization method we describe could be used in a larger, prospective study, that would also assess performance as observers gain experience with color coded images.

### Limitations

Color-based visualization is more technically challenging than grayscale visualization. For example, the calibration of color displays is more laborious as the calibration of grayscale displays – and not as widely used^2^. However, the extra effort for calibration of color displays in medical imaging might be justified by other benefits of color-coding information.

Another limitation of color-based methods is related to the observer: Color vision anomalies are quite common and might compromise diagnostic reliability of these methods. Especially, 8% of the male population and 0.4% of the female population have detectable color vision abnormalities, most commonly deuteranomaly^26^,^27^,^28^. For these observers, the color maps we present are probably not optimally suited.

### Visualization versus automatic classification of multiparametric data sets

Human observers cannot use an arbitrary number of information channels for their decision-making process. Consequently, in practice, only a small number of different MR measurements is used to make a diagnosis. Computer-based classification algorithms are a way to overcome this limitation, because they can combine an arbitrary number of measurements to a single metric. Automatic tissue classification methods have been established for histological imaging^29^,^30^, CT imaging^31^, and multivariate MR imaging, e.g. for the detection of cancer in breast^32^ and prostate cancer^33^. Especially, such a classification method has been successfully applied for automatic prostate cancer grading from mpMRI data^34^. Automatic methods for the detection of clinically relevant pathologies are also summarized as “*computer-aided diagnosis*”^35^. These approaches have been successfully combined with human decision making, thereby complementing, instead of substituting, human observers^36^. More recently, fully automatic approaches (termed *radiomics*) have been used to extract even more clinically useful information from imaging datasets^37^,^38^. These approaches use raw data and do not require any human interaction.

Still, even today, visual examination by a trained human observer remains the gold standard in diagnostic imaging. Although machine learning algorithms can incorporate a huge number of parameters in their computations, humans still outperform automatic algorithms in many visual pattern recognition tasks in medicine. There are several possible reasons for this – and one of the main reasons is that humans excel in combining heterogeneous data from different sources. A clinical radiologist considers much more information than just the raw image data: he or she considers patient information (age, sex, lifestyle, family history), previous diseases, previous surgery, previous imaging studies, laboratory tests such as prostate-specific antigen (PSA) and other factors.

Given that humans are very good at deriving decisions from data, it is a relevant problem how to make this data accessible to them. In an medical imaging context, making data accessible relates to optimally displaying imaging data, especially high-dimensional MR information so that human observers can make a well-informed clinical decision. Potentially, the method we present here is a powerful tool to achieve this.

## Additional Information

**How to cite this article**: Kather, J. N. *et al*. Color-coded visualization of magnetic resonance imaging multiparametric maps. *Sci. Rep.* **7**, 41107; doi: 10.1038/srep41107 (2017).

**Publisher's note:** Springer Nature remains neutral with regard to jurisdictional claims in published maps and institutional affiliations.

## Figures and Tables

**Table 1:** MRI parameters for prostate imaging.

	T2 axial	DWI	DCE
Sequence	TSE	EPI DWI	2D FLASH
TR (ms)	6360	1300	193
TE (ms)	108	67	1.05
FOV (mm^2^)	160 × 160	200 × 200	300 × 225
Voxel size (mm^3^)	0.7 × 0.6 × 3	2.2 × 2.2 × 4	2.1 × 1.6 × 6
Matrix	256 × 230	90 × 90	192 × 144
PAT factor	2	—	—
Number of slices	22	10	4–6
B-value	—	0,50,150,300,600,800	—
No. of measurements	1	1	50
Temporal resolution (sec)	—	—	4

DWI = diffusion weighted imaging, DCE = dynamic contrast enhanced, FLASH = fast low angle shot, TE = echo time, TR = repetition time, TSE = turbo spin echo, PAT = parallel imaging factor.

**Table 2:** Clinico-pathological characteristics of the prostate sample collective.

Pseudonym	Age	Gleason	TNM, L, V, R
112	69	4 + 4 = 8	pT3b pNx pMx L1 V1 R0
121	63	4 + 5 = 9	pT3b pN1 pMx L1 V1 R1
152	59	3 + 4 = 7	pT2c pN0 pMx L0 V0 R0
159	72	4 + 3 = 7	pT3b pNx pMx L0 V0 R0
186	52	3 + 3 = 6	pT2c pNx pMx L0 V0 R0
187	59	3 + 4 = 7	pT2c pNx pMx L0 V0 R1
225	56	4 + 3 = 7	pT2c pN0 pMx L0 V0 R0
226	59	3 + 4 = 7	pT2c pN0 pMx V0 L0 R0
227	67	3 + 3 = 6	pT2c pNx pMx L0 V0 R0
234	69	3 + 4 = 7	pT2b pN0 pMx L1 V0 R0
237	68	3 + 3 = 6	pT2c pN0 pMx L0 V0 R0
238	73	3 + 4 = 7	pT3a pNx pMx L0 V0 R0
260	58	3 + 4 = 7	pT3a pN0 pMx L0 V0 R0

**Table 3:** Sensitivity (Sens.), Specificity (Spec.), Positive/Negative Predictive Value (PPV/NPV) for each observer in our prostate cancer detection study.

	Sens.	Spec.	PPV	NPV
Radiologist expert gray	0.36	0.92	0.77	0.65
Radiologist 1 color	0.19	0.92	0.64	0.60
Radiologist 2 color	0.26	0.90	0.66	0.61
Radiologist 3 color	0.15	0.95	0.69	0.59
Radiologist 4 color	0.17	0.96	0.75	0.60
Radiologists color mean	0.21	0.95	0.76	0.61
Radiologist expert color	0.36	0.91	0.75	0.65

**Table 4:** Contingency table for the presence of prostate cancer in each segment of the prostate, given for each observer in our study.

	Pathologist pos.	Pathologist neg.
Radiologist expert gray pos.	64	19
Radiologist expert gray neg.	116	217
Radiologist 1 color pos.	35	20
Radiologist 1 color neg.	145	216
Radiologist 2 color pos.	47	24
Radiologist 2 color neg.	133	212
Radiologist 4 color pos.	27	12
Radiologist 4 color neg.	153	224
Radiologist 5 color pos.	30	10
Radiologist 5 color neg.	150	226
Radio. mean color pos.	37	12
Radio. mean color neg.	143	224
Radio. expert color pos.	64	21
Radio. expert color neg.	116	215
